# Impact of integrated district level mental health care on clinical and social outcomes of people with severe mental illness in rural Ethiopia: an intervention cohort study

**DOI:** 10.1017/S2045796019000398

**Published:** 2019-08-13

**Authors:** C. Hanlon, G. Medhin, M. Selamu, R. Birhane, M. Dewey, K. Tirfessa, E. Garman, L. Asher, G. Thornicroft, V. Patel, C. Lund, M. Prince, A. Fekadu

**Affiliations:** 1 Health Service and Population Research Department, King's College London, Institute of Psychiatry, Psychology and Neuroscience, Centre for Global Mental Health, London, UK; 2 WHO Collaborating Centre for Mental Health Research and Capacity Building, Department of Psychiatry, Addis Ababa University, College of Health Sciences, School of Medicine, Addis Ababa, Ethiopia; 3 Centre for Innovative Drug Development and Therapeutic Trials for Africa (CDT-Africa), College of Health Sciences, Addis Ababa University, Addis Ababa, Ethiopia; 4 Addis Ababa University, Aklilu Lemma Institute of Pathobiology, Addis Ababa University, Addis Ababa, Ethiopia; 5 Kotebe Metropolitan University, College of Education and Behavioral Studies, Addis Ababa, Ethiopia; 6 Department of Psychiatry and Mental Health, Alan J Flisher Centre for Public Mental Health, University of Cape Town, Cape Town, Republic of South Africa; 7 Division of Epidemiology and Public Health, University of Nottingham, School of Medicine, Nottingham, UK; 8 Department of Global Health and Social Medicine, Harvard Medical School, Boston, USA; 9 Department of Global Health and Population, Harvard TH Chan School of Public Health, Boston, USA; 10 Sangath Non-Governmental Organisation, Goa, India; 11 Global Health & Infection Department, Brighton and Sussex Medical School, Brighton, UK

**Keywords:** Bipolar disorder, community mental health, global mental health, psychotic disorder, schizophrenia, task-sharing

## Abstract

**Aim:**

There is limited evidence of the safety and impact of task-shared care for people with severe mental illnesses (SMI; psychotic disorders and bipolar disorder) in low-income countries. The aim of this study was to evaluate the safety and impact of a district-level plan for task-shared mental health care on 6 and 12-month clinical and social outcomes of people with SMI in rural southern Ethiopia.

**Methods:**

In the Programme for Improving Mental health carE, we conducted an intervention cohort study. Trained primary healthcare (PHC) workers assessed community referrals, diagnosed SMI and initiated treatment, with independent research diagnostic assessments by psychiatric nurses. Primary outcomes were symptom severity and disability. Secondary outcomes included discrimination and restraint.

**Results:**

Almost all (94.5%) PHC worker diagnoses of SMI were verified by psychiatric nurses. All prescribing was within recommended dose limits. A total of 245 (81.7%) people with SMI were re-assessed at 12 months. Minimally adequate treatment was received by 29.8%. All clinical and social outcomes improved significantly. The impact on disability (standardised mean difference 0.50; 95% confidence interval (CI) 0.35–0.65) was greater than impact on symptom severity (standardised mean difference 0.28; 95% CI 0.13–0.44). Being restrained in the previous 12 months reduced from 25.3 to 10.6%, and discrimination scores reduced significantly.

**Conclusions:**

An integrated district level mental health care plan employing task-sharing safely addressed the large treatment gap for people with SMI in a rural, low-income country setting. Randomised controlled trials of differing models of task-shared care for people with SMI are warranted.

## Introduction

The treatment gap for people with severe mental illness (SMI) is over 90% in most low-income countries (Wang *et al*., 2007). A ‘full’ task-sharing model whereby primary healthcare (PHC) workers are equipped to deliver all aspects of first-line mental health care, with limited specialist support, is recommended to increase access to care. Full task-sharing is at the heart of the World Health Organisation (WHO)'s mental health Gap Action Programme (mhGAP) (World Health Organization, 2008). However, many studies of task-shared care for SMI have employed a model whereby a mental health specialist makes the diagnosis of SMI, initiates treatment and provides an ongoing review, combined with the involvement of non-specialists in delivering psychosocial aspects of care (Chatterjee *et al*., 2014). This ‘partial’ model of task-sharing cannot address the needs of most people with SMI in low-income countries due to the scarcity of mental health professionals. In the few examples of programmes employing a ‘full’ task-sharing approach (Ventevogel *et al*., 2012; Gureje *et al*., 2015), there is only limited evidence on whether care can be delivered safely and with sufficient quality to bring about improved outcomes for people with SMD (Jordans *et al*., 2017; Jordans *et al*., 2019). This lack of evidence may be fuelling reluctance to embrace the more ambitious task-sharing approach (Hanlon *et al*., 2016*b*). To address this evidence gap, we present findings from the Programme for Improving Mental health carE (PRIME) (Fekadu *et al*., 2016). We have shown that PRIME in Ethiopia achieved contact coverage of over 80% for people with SMI in the target population. The objective of the current study was to investigate the impact of implementing a district level mental health care plan on the 12-month clinical and social outcomes of people with SMI who engaged with primary mental health care in Ethiopia.

## Methods

### Study design

We conducted an intervention cohort study, with assessments conducted at the baseline of implementation of the district mental health care plan (T0), and 6 (T1) and 12 months (T2) after initial engagement of people with SMI in the new integrated service.

### Setting

The PRIME study was carried out in Sodo district, in the Gurage Zone of the Southern Nations, Nationalities and Peoples' Region of southern Ethiopia from December 2014 to July 2016. Sodo has an estimated population of around 160 000 people (Hanlon *et al*., 2014). More than 90% of inhabitants live in rural areas and rely on subsistence farming and small-scale trading. At the time of the study, health services in Sodo comprised 54 health posts, most of which were staffed by two community health extension workers with 1 year of training in general health promotion and illness prevention, and eight health centres, staffed by nurses, health officers and midwives, who provide basic curative and obstetric care. There were no doctors or mental health specialists in the district. In the nearest town of Butajira, located 30–50 km from Sodo, there was a psychiatric nurse-led out-patient clinic. The nearest in-patient mental health services were 100–120 km away in the capital city, Addis Ababa.

### Sample and recruitment procedures

As described previously (Baron *et al*., 2018), people with possible SMI in the community were identified by community key informants and health extension workers who had received half a day of training in typical presentations of SMI (Shibre *et al*., 2002). People with possible SMI were referred to the nearest health centre and assessed by PHC workers who had been trained in mental health care for a total of 10 days: 5 days of classroom-based teaching using adapted mhGAP training materials and 5 days of practical clinical training in the Butajira psychiatric clinic. The PHC workers assessed the referrals, made a diagnosis of SMI and initiated treatment according to the evidence-based clinical guidelines in the WHO mhGAP Intervention Guide (World Health Organization, 2016). Independent diagnostic review was carried out with standardised, semi-structured clinical assessments by research psychiatric nurses using the OPerational CRITeria for research (OPCRIT) (McGuffin *et al*., 1991). Diagnostic assessments were conducted for all people with PHC worker-diagnosed SMI and wherever the PHC worker was uncertain about the diagnosis. The research psychiatric nurses reviewed the initial treatment plan. Any changes made by the psychiatric nurses were communicated back to the PHC workers.

### Eligibility criteria

- Confirmatory research psychiatric nurse diagnosis of a psychotic disorder (including schizophrenia, schizoaffective disorder and depression with psychotic features) or bipolar disorder.
- Providing informed consent, or caregiver permission, to participate in the study if the person with SMI lacked capacity to consent.
- Able to converse in Amharic, the official language of the region.
- Planning to reside in the district for at least 12 months.
- No cognitive or sensory impairment that interfered substantially with the clinical assessment.
- Not acutely physically unwell.

### Sample size

The sample size for the PRIME SMI cohorts across countries was calculated to detect a 20% reduction in the severity of symptoms at 12 months, with 90% power, two-sided alpha of 0.05 and 20% attrition rate (Baron *et al*., 2018), leading to a target sample size of 150. However, in the Ethiopia district, all people who received a confirmatory diagnosis of SMI were included to establish a completely ascertained population cohort.

### PRIME intervention for people with SMI

The integrated district mental health care plan for the Ethiopian setting has been described in detail previously (Fekadu *et al*., 2016), and involved interventions at the level of the health system, PHC facility and community.

#### Health system interventions

The district health office staff were involved in participatory planning using Theory of Change methodology (Hailemariam *et al*., 2015). The district health office assigned a focal person for mental health co-ordination. PRIME provided ongoing technical support with medication supply management (including establishing a revolving drug fund and providing assistance with forecasting the amount of psychotropic medication required), building capacity in supervision of mental healthcare (training high-performing general health workers to supervise mental health care) and monitoring and evaluation activities (e.g. aggregating data on facility contacts and feeding back at the advisory board meetings).

#### PHC facility interventions

All frontline PHC workers in Sodo district (*n*  =  128) were trained in mental healthcare. The PHC workers received monthly supervision from a psychiatric nurse trained using the mhGAP supervisor training manual. For rural health centres, supervision was conducted by telephone when weather conditions precluded travel to the facility. PHC workers could also consult the psychiatric nurse for advice. People with confirmed SMI were prescribed an antipsychotic and/or antidepressant medication and/or a benzodiazepine, as indicated. There were no mood-stabiliser medications available, so people with bipolar disorder received antipsychotic medication as per usual practice in this setting (Fekadu *et al*., 2015). Almost all service users had to pay for medication.

In addition to the prescription of medication, PHC workers were trained to provide psychoeducation, activate social supports, address social stressors, monitor physical health, review response to treatment and refer to specialist mental health care if needed. As prescriptions were usually for a maximum of 1 month, follow-up appointments with the PHC workers were usually scheduled monthly.

#### Community-level interventions

A multi-sectoral ‘community advisory board’ was established to support community awareness-raising and mobilisation, to help with trouble-shooting during the implementation phase and to review project activities and outcomes. A total of 96 community-based health extension workers were trained in case detection, outreach to re-engage people who dropped out of care, identification of medication side effects, community awareness-raising and supporting social reintegration and recovery of people with SMI. In half of the sub-districts, people with schizophrenia who had enduring symptoms or disability after 6 months received adjuvant community-based rehabilitation, delivered by trained lay workers (*n*  =  75) or ongoing PRIME care (*n*  =  87), as part of a nested cluster randomised trial (the RISE trial) (Asher *et al*., 2016). Aside from the additional contacts from CBR workers in the intervention arm (weekly for 2–3 months and 2-weekly for the subsequent 5–6 months), there were no additional trial-related contacts.

### Measures

#### Primary outcomes

- Clinical symptom severity was measured using the Brief Psychiatric Rating Scale, expanded version (BPRS-E) (Burlingame *et al*., 2006). The BPRS-E is a 24-item, clinician-rated scale which has been translated into Amharic and shown to have robust psychometric properties and sensitivity to change in Ethiopia (Habtamu *et al*., 2017).
- Disability was measured using the World Health Organisation Disability Assessment Schedule (WHODAS), version 2.0, 36-item version (Üstün *et al*., 2010). The WHODAS has been validated for use in people with SMI in Ethiopia and is sensitive to change (Habtamu *et al*., 2017). The WHODAS was completed by a combination of responses from the person with SMI and the caregiver responses to the proxy-WHODAS at the post-baseline assessments (19.8% of WHODAS scores from caregivers at midline, 24.1% at endline). We used the polytomous summary score of the WHODAS scaled from 0 to 100.

#### Secondary outcomes

- Experience of discrimination was measured using the ‘unfair treatment’ subscale of the discrimination and stigma scale-12 (DISC-12) (Brohan *et al*., 2013). The original DISC-12 sub-scale has 21 items. Four items lacked face validity or had a low frequency of endorsement, but the remaining 17 items loaded onto a single factor using exploratory factor analysis and were summed.
- Restraint was measured by self-report of whether the person had been ‘restrained, chained or confined’ in the preceding 12 months.
- Alcohol use disorder was measured using the lay interviewer-administered Alcohol Use Disorder Identification Test (AUDIT) (Babor *et al*., 2001). This 10-item scale has been adapted for local drinks in the Ethiopian setting. People scoring ⩾8 are considered to have a probable alcohol use disorder.
- Depression was measured using a locally validated version of the Patient Health Questionnaire (PHQ-9) (Kroenke and Spitzer, 2002). In the Ethiopian setting, a cut-off of 5 or more is indicative of major depressive disorder (Hanlon *et al*., 2015).
- Suicide attempts in the past 3 months were assessed using the Mini International Neuropsychiatric Interview (Sheehan *et al*., 1997).

### Potential effect modifiers

#### Equity indicators

- Gender and residence (rural *v*. urban).
- Socio-economic status: a poverty index was constructed which loaded onto a unidimensional scale using exploratory factor analysis: roof material made of straw (*v*. corrugated iron), unimproved water source, unimproved sanitation, no electricity, no separate room for kitchen, no radio or television, no mobile phone.
- Time to access the nearest health facility, estimated in minutes, whatever the means of travel. This was dichotomised into <60 min *v*. 60 min or longer.

#### Baseline characteristic

- Diagnosis: clinician-assessed diagnosis using criteria of the Diagnostic and Statistical Manual of the American Psychiatric Association, version IV) (American Psychiatric Association, 1994) from OPCRIT (McGuffin *et al*., 1991).

#### Process indicators

- Receipt of minimally adequate treatment used the definition proposed by Wang *et al*. (Wang *et al*., 2002): prescription of medication on at least one occasion combined with at least four follow-up appointments with a health worker trained in mental health. We additionally required that psychotropic medication should be prescribed at therapeutic levels (World Health Organization, 2016). Data on the number of facility contacts and prescriptions were extracted from the clinical records, cross-referenced with a facility registration book.
- Receipt of community support (measured at T1 and T2): support received with returning to work, remembering to take medication, improving self-care, meeting people and social engagement. Responses were summed and binarised: 0–2 types of community support *v*. 3–5 types of community support.
- Receipt of in-patient care for mental health problems, contact with specialist mental health or general health facilities and traditional or religious healers, and type of care received during primary health care contacts were measured at T1 and T2.

### Descriptive baseline characteristics

- Socio-demographic characteristics (age, educational level, marital status).
- Social support was assessed using the three-item Oslo Social Support Scale (OSS-3) (Dalgard *et al*., 2006), which asks about number of close supports, extent of concern from supports and amount of practical support received. The OSS-3 total score was categorised as follows: 3–8 ‘poor support’, 9–11 ‘intermediate support’ and 12–14 ‘strong support’.
- Duration of illness, type of illness onset (acute/sub-acute *v*. gradual), psychiatric hospitalisation in the past 12 months, presence of co-morbid medical condition and receipt of psychotropic medication at baseline were obtained from the OPCRIT (McGuffin *et al*., 1991).

### Data collection

The lay interviewers were individuals with an educational level of at least tenth grade who were recruited from the local area and trained for 12 days on the study questionnaires and protocols, including observed practice interviews. The trainers had master's level qualifications. Degree-level supervisors monitored data quality in the field. The clinician assessments were conducted by research psychiatric nurses who were trained for 7 days by senior Ethiopian psychiatrists. The OPCRIT diagnoses were double-checked by an Ethiopian psychiatrist by reviewing the OPCRIT responses and clinical documentation and conducting verification interviews (*n*  =  2) where needed.

### Data management and analysis

Data were double-entered using EpiData (Lauritsen and Bruus, 2003) and analysed using Stata version 13.1 (StataCorp LP, 2016). The data were summarised descriptively, with outcome data stratified by equity indicators (gender, residence, distance from the health facility and poverty status). Comparison of the characteristics of participants remaining in the cohort with those lost to follow-up at 12 months, as well as by equity indicators, was conducted using Pearson chi-squared for categorical variables, Student's *t*-test for comparing means in normally distributed variables and Kruskal Wallis equality-of-populations rank test for non-normal continuous variables.

Mixed effects linear regression with random intercept was used to model the change in symptom severity, disability and depressive symptoms over time. We tested for improvement in model fit using likelihood ratio tests after adding random slopes. We also tested for any significant difference in the mean change between T0 and T1 or between T1 and T2. Mixed effects ordinal regression was used to model change in discrimination score over time. A prevalence ratio was calculated for change from T0 to T2 in probable alcohol use disorder (AUDIT ⩾ 8), suicide attempt in the preceding 3 months or restraint in the preceding 12 months.

For the primary outcomes, we calculated standardised mean difference (Borenstein *et al*., 2009) and examined effect modification by the equity indicators, diagnosis (primary psychotic disorder *v*. affective disorder), process indicators (receipt of minimally adequate treatment and community support) and by the community-based rehabilitation intervention group for the RISE trial. This was done by adding an interaction term into the model and testing for improved model fit using a likelihood ratio test. Multiple linear regression analysis was conducted to examine the association between baseline duration of illness and type of illness onset with 12-month outcomes.

### Ethical considerations

Ethical approval was obtained from the Institutional Review Board of the College of Health Sciences, Addis Ababa University (No. 084/11/Psy). Informed consent was obtained where possible. If the person lacked capacity and did not refuse, the accompanying caregiver was invited to give permission on the person's behalf.

## Results

**Fig. 1.**
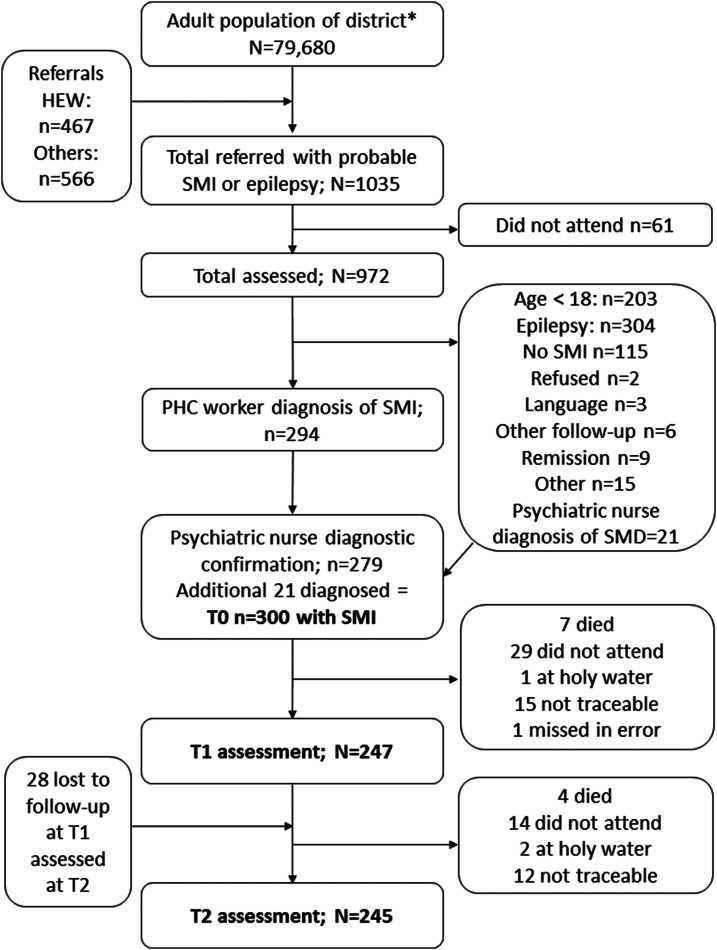
Flow diagram.

Of the 294 people diagnosed by PHC workers as having SMI, 279 were confirmed to have SMI, giving a positive predictive value for PHC worker diagnosis of 94.9%. A further 21 people referred by PHC workers to psychiatric nurses for diagnostic review were also found to have SMI, giving a total of 300 participants. See Fig. 1.

### Baseline characteristics

See Table 1. At baseline, there was no significant difference in symptom severity score, disability, discrimination or depressive symptoms by gender. The proportion with an alcohol use disorder (*χ*^2^(1) 48.6750; *p* < 0.001) or who had been restrained (*χ*^2^(1) 5.1154; *p*  =  0.024) was higher in men. Baseline disability (*t*  =  2.1012; *p*  =  0.04) and restraint (*χ*^2^(1) 3.9058; *p*  =  0.048) were significantly higher in those with low socioeconomic status. Perceived negative discrimination at baseline was higher in urban residents (*χ*^2^(1) 3.986; *p*  = 0.049) and people of low socioeconomic status (*χ*^2^(1) 4.195; *p*  =  0.04).

**Table 1.** Baseline characteristics of the severe mental disorder cohort stratified by gender

Characteristics	Total *N* (%)	Male *N* (%)	Female *N* (%)
	300 (100.0)	172 (57.3)	128 (42.7)
Age (years)	Mean (s.d.)	35.5 (13.45)	36.6 (12.86)	34.1 (14.1)
Educational level (*n* = 299)	No formal education	157 (52.5)	79 (46.2)	78 (60.9)
	Primary education	112 (37.5)	75 (43.9)	37 (28.9)
	Secondary and above	30 (10.0)	17 (9.9)	13 (10.2)
Religious affiliation	Orthodox Christian	270 (90.0)	154 (89.5)	116 (90.6)
	Muslim	10 (3.3)	7 (4.1)	3 (2.3)
	Protestant Christian	19 (6.3)	10 (5.8)	9 (7.0)
	None	1 (0.3)	1 (0.6)	0 (0.0)
Ethnicity	Gurage	284 (94.7)	162 (94.2)	122 (95.3)
	Other	16 (5.3)	10 (5.8)	6 (4.7)
Marital status	Married	111 (37.0)	66 (38.4)	45 (35.2)
	Single	136 (45.3)	83 (48.3)	53 (41.1)
	Divorced or widowed	53 (17.7)	23 (13.4)	30 (23.4)
Household size (*n* = 298)	1–2	34 (11.4)	19 (11.1)	15 (11.8)
	3–4	85 (28.5)	46 (26.9)	39 (30.7)
	5–6	92 (30.9)	47 (27.5)	45 (35.4)
	7 or more	87 (29.2)	59 (34.5)	28 (22.1)
Children in household (*n* = 287)	None	138 (48.1)	89 (54.3)	49 (39.8)
	⩽5 years	67 (23.3)	33 (20.1)	34 (27.6)
	5–15 years	58 (20.2)	35 (21.3)	23 (18.7)
	16 years and older	24 (8.4)	7 (4.3)	17 (13.8)
Socio-economic status (*n* = 297)	Higher (poverty index* ⩽ 3)	177 (59.6)	100 (58.5)	77 (61.1)
	Lower (poverty index > 3)	120 (40.4)	71 (41.5)	49 (38.9)
Residence (*n* = 299)	Urban	60 (20.1)	36 (20.9)	24 (18.9)
	Rural	239 (79.9)	136 (79.1)	103 (81.1)
Social support (*n* = 298)	Strong social support	56 (18.8)	31 (18.2)	25 (19.5)
	Intermediate support	151 (50.7)	83 (48.8)	68 (53.1)
	Poor support	91 (30.5)	56 (32.9)	35 (27.3)
Travel time to nearest health facility (*n* = 299)	⩽60 min	192 (64.2)	115 (67.3)	77 (60.2)
	61–120 min	60 (20.1)	32 (18.7)	28 (21.9)
	⩾121 min	47 (15.7)	24 (14.0)	23 (18.0)
Diagnosis	Affective psychosis/bipolar disorder	44 (14.7)	21 (12.2)	23 (18.0)
	Schizophrenia & other psychoses	256 (85.3)	151 (87.8)	105 (82.0)
Duration of illness (years) (*n* = 270)	Median (interquartile range; IQR)	5 (2.8–10)	5 (2.8–10)	5.5 (2.8–11)
Onset of illness (*n* = 271)	Acute/sub-acute	100 (36.9)	55 (35.5)	45 (38.8)
	Gradual	171 (63.1)	100 (64.5)	71 (61.2)
Psychiatric admission	In past 12 months	11 (3.7)	5 (2.9)	6 (4.7)
Treatment at recruitment (*n* = 261)	Prescribed medication at baseline	69 (26.4)	35 (23.5)	34 (30.4)
Co-morbid medical disorder (*n* = 286)	Diagnosed medical condition	18 (6.3)	13 (8.1)	5 (4.0)

*Poverty index  = roof material made of straw (*v*. corrugated iron), unimproved water source, unimproved sanitation, no electricity, no separate room for kitchen, no radio or television, no mobile phone.

### Cohort follow-up

A total of 247 (82.3%) people were assessed at T1 (mean 7.4 months; s.d. 1.49) and 245 (81.7%) at T2 (mean 12.3 months; s.d. 1.12) (see Fig. 1). There was no evidence of differential loss to follow-up based on baseline characteristics (online Supplementary File 1). During the follow-up period, 11 participants died.

### Facility-based intervention

After review by psychiatric nurses, the medication initiated by PHC workers was unchanged or changed within the same medication class for 184 (67.7%) participants. Psychiatric nurses increased the dose or added another medication for 46 (16.9%), stopped or reduced the dose in 15 (5.9%), changed the class of medication in 15 (5.5%) and stopped a prescription of depot antipsychotic medication in 5 (1.8%).

Participants attended a median of two PHC appointments during the follow-up period (IQR 2–4; minimum 1 and maximum 12) (online Supplementary File 2). Minimally adequate treatment was received by 89 (29.8%) (online Supplementary File 3). There was no evidence of prescribing above recommended limits and only one occurrence of antipsychotic polypharmacy. Admission for in-patient care was very low (1.4% at T1 and 2.0% at T2) and <10% of participants had direct contact with a mental health specialist. A high proportion reported receiving psychosocial support and explanation about medication (online Supplementary File 4).

### Community-based interventions

Most people with SMI reported receiving support with medication adherence and to improve self-care. More than half received support to get back to work, but less than a quarter were supported to get involved in social activities and <10% had support with meeting people. Almost all support was reported to come from the family. A small proportion reported contact with traditional or religious healers: 13.0% at T1 and 11.6% at T2.

### Impact on clinical and social outcomes

There was a significant improvement in all clinical (symptom severity score, depressive symptoms, suicide attempts, alcohol use disorder) and social (functioning, discrimination, restraint) (Tables 2 and 3). The standardised mean difference for symptom severity was 0.18 (95% confidence interval (CI)) 0.02–0.34) between T0 and T1 and 0.28 (95% CI 0.13–0.44) from T0 to T2. For disability, the standardised mean difference was larger at both time-points: T0–T1: 0.27 (95% CI 0.13–0.41), T0–T2: 0.50 (95% CI 0.35–0.65). The test for interaction between the RISE trial intervention group and the main outcomes (symptom severity and functioning) at 12 months was non-significant.

**Table 2.** Mixed-effects modelling of primary outcomes stratified by equity indicators

Outcome		Baseline (T0) Mean (s.d.)	T0–T1 mean difference (95% confidence intervals	T0–T2 mean difference (95% confidence intervals
**SMI symptoms (BPRS-E)**		*N* = 294	*N* = 247	*N* = 245
Total sample		48.5 (15.6)	**−2.6 (−4.8** to **−0.4)**	**−4.8 (−7.0** to **−2.6)**
Gender	Male	47.9 (16.3)	−2.2 (−5.2 to 0.9)	**−3.9 (−6.9** to **−0.8)**
	Female	49.4 (14.7)	**−3.2 (−6.3** to **−0.04)**	**−6.0 (−9.1** to **−2.8)**
Residence	Urban	47.4 (16.5)	−4.0 (−9.3 to 1.2)	**−9.0 (−14.2** to **−3.8)**
	Rural	48.9 (15.4)	−2.3 (−4.7 to −0.1)	**−4.1 (−6.5** to **−1.7)**
Health care access	<60 min	48.2 (16.2)	**−2.1 (−4.8** to **−0.7)**	**−5.5 (−8.3** to **−2.8)**
	⩾60 min	49.3 (15.6)	**−3.9 (−7.5** to **−0.3)**	−3.4 (−7.0 to 0.3)
Socio-economic status	Higher	49.2 (16.0)	**−3.5 (−6.6** to **−0.5)**	**−5.6 (−8.6** to **−2.5)**
	Lower	47.4 (15.1)	−0.8 (−3.9 to 2.4)	**−3.1 (−6.2** to **−0.02)**
**Disability (WHODAS 2.0)**		*N* = 296	*N* = 246	*N* = 245
Total sample		52.2 (22.0)	**−6.2 (−9.3** to **−3.1)**	**−12.0 (−15.1** to **−8.9)**
Gender	Male	52.3 (21.5)	**−7.8 (−11.8** to **−3.8)**	**−10.1 (−14.2** to **−6.0)**
	Female	52.1 (22.7)	−4.2 (−9.0 to 0.6)	**−14.3, (−19.1** to **−9.6)**
Residence	Urban	49.2 (22.3)	**−7.4 (−14.0** to **−0.7)**	**−14.1 (−20.6** to **−7.5)**
	Rural	53.1 (21.9)	**−6.2 (−9.7** to **−2.7)**	**−11.8 (−15.3** to **−8.2)**
Health care access	<60 min	50.4 (22.5)	**−4.8 (−8.7** to **−0.8)**	**−11.4 (−15.3** to **−7.4)**
	⩾60 min	55.9 (20.7)	**−8.9 (−13.9** to **−3.8)**	**−13.4 (−18.5** to **−8.3)**
Socio-economic status	Higher	50.0 (21.8)	**−6.9 (−11.1** to **−2.7)**	**−11.4 (−15.6** to **−7.2)**
	Lower	55.3 (22.1)	**−4.7 (−9.3** to **−0.1)**	**−12.4 (−17.0** to **−7.9)**

The figures in bold are significant at *p* < 0.05.

**Table 3.** Secondary clinical and social outcomes in people with SMI stratified by gender

Outcome	Baseline (T0)	Follow-up (T2)	T0–T2 change
	Mean (s.d.)	Mean (s.d.)	Mean difference
Depression symptoms (PHQ-9)	*N* = 300	*N* = 245	
Total sample	12.8 (5.43)	7.2 (4.80)	**−5.6 (−6.3** to **−4.8)**
Male	12.9 (5.64)	7.4 (4.84)	**−5.4 (−6.5** to **−4.4)**
Female	12.8 (5.15)	7.0 (4.75)	**−5.7 (−6.8** to **−4.6)**
	**Median (IQR)**	**Median (IQR)**	**Odds ratio**
Discrimination (DISC-12)	*N* = 300	*N* = 245	
Total sample	2 (0–7)	0 (0–3)	**0.4 (0.3–0.5)**
Male	2 (0–7)	0 (0–4)	**0.5 (0.3–0.8)**
Female	3 (0–7)	0 (0–2)	**0.2 (0.1–0.4)**
	***N* (%)**	***N* (%)**	**Prevalence ratio**
Alcohol use disorder (AUDIT ⩾ 8)	*N* = 300	*N* = 245	
Total sample	87 (29.0)	40 (16.3)	**0.6 (0.5–0.8)**
Male	77 (44.8)	34 (25.2)	**0.6 (0.4–0.8)**
Female	10 (7.8)	6 (5.5)	0.7 (0.3–1.4)
Suicide attempts past 3 months	*N* = 300	*N* = 245	
Total sample	42 (14.0)	12 (4.9)	**0.3 (0.2–0.6)**
Male	20 (11.6)	8 (5.9)	0.5 (0.2–1.0)
Female	22 (17.2)	4 (3.6)	**0.2 (0.1–0.5)**
Restrained past 12 months	*N* = 300	*N* = 245	
Total sample	76 (25.3)	26 (10.6)	**0.4 (0.3–0.6)**
Male	52 (30.2)	13 (9.6)	**0.3 (0.2–0.5)**
Female	24 (18.8)	13 (11.8)	**0.6 (0.3–1.0)**

The figures in bold are significant at *p* < 0.05.

For symptom severity and functioning, the magnitude of the change did not differ between T0 and T1 compared to T1 and T2. The reduction in depressive symptoms was significantly greater between T0 and T1 than between T1 and T2 (*χ*^2^(1) 4.26; *p*  =  0.039); similarly for change in perceived discrimination (*χ*^2^(1) 3.75; *p*  =  0.053).

There was no statistically significant effect modification by the equity indicators, diagnosis or process indicators for the primary outcomes. At T1, higher receipt of community support had a borderline statistical association with a greater reduction in symptom severity (*p*  =  0.19) and disability (*p*  =  0.09) (online Supplementary Files 5 and 6). There was no association between duration of illness or type of illness onset and the mean improvement in the primary outcomes.

## Discussion

In this community-ascertained intervention cohort of clinician-confirmed people with SMI, there was a significant improvement in clinical and social outcomes after implementation of a district level mental health care plan. PHC workers diagnosed SMI accurately, prescribed psychotropic medication safely and were reported to have delivered psychoeducation and provided support to most people with SMI. The findings from this study are generalisable to similar rural settings of low-income countries.

Although PHC workers prescribed safely (low polypharmacy and no doses above the recommended therapeutic range), the psychiatric nurses did consider that it was necessary to change the initial prescriptions of psychotropic medication in 30.1% of cases. This reinforces the need for task-shared care to be supported by input by mental health specialists, either through regular supervision or through timely review of newly diagnosed cases.

We are only aware of two previous studies, both from Nepal, where the impact of a ‘full’ task-sharing model on clinical and social outcomes of people with SMI was evaluated (Jordans *et al*., 2017; Jordans *et al*., 2019). Both studies found a significant reduction in symptom severity, disability and caregiver burden but had sample sizes under 100 and included extensive community-based psychosocial interventions (Jordans *et al*., 2019). Our larger study, which included a more representative population of people with SMI and a task-sharing intervention more closely based on mhGAP, provides more definitive evidence of important clinical and social benefits from the recommended WHO mhGAP approach.

In our study, the impact of the district mental health care plan care on psychotic symptoms was less marked than the reductions seen in the social outcomes, with a reduction of 4.8 on the BPRSE being less than that usually considered to be clinically significant (Hanlon *et al*., 2016*a*). In previous intervention studies for people with SMI in LMIC settings, a key factor for clinical improvement has been enhanced adherence to antipsychotic medication (Chatterjee *et al*., 2014), but only 30% of people with SMI in our study received ‘minimally adequate treatment’ over the follow-up period. Our process data indicate that engagement waxed and waned over time, rather than people dropping out of care altogether, and lends support to the acceptability of care provided as well as providing explanation for the limited impact on symptom improvement at cross-sectional assessment. In-depth interviews with study participants with SMI who had disengaged from care indicated that most had experienced symptomatic improvement, but that affordability of medication and side effects of medications was a barrier to continuous engagement. This is supported by quantitative data from the same sample, indicating high levels of poverty and out-of-pocket healthcare costs compared to the general population (Hailemichael *et al*., 2019). Mechanisms to reduce out-of-pocket healthcare costs for people with SMI are needed to achieve improved access to mental health care (Hanlon *et al*., 2019).

Although disability is closely linked to symptom severity, our previous work in this community indicates that stigma, discrimination and poverty also make important contributions. The borderline significant effect modification indicating greater improvements in disability in people with higher receipt of community support may reflect the impact of reduced social exclusion. We observed a significant decline in perceived negative discrimination in people with SMI. The PRIME mental health care plan included a cascade model of training of community-based health extension workers to raise community awareness and reduce stigma against mental health problems (Fekadu *et al*., 2016); however, the extent of implementation is not known. The growing community and family awareness of the treatability of SMI, arising from the local availability of a treatment service and bearing witness to the clinical improvement of people with SMI who were well-known to the community, might have also reduced stigmatising attitudes, social exclusion and the need for people to be restrained. Furthermore, the Community Advisory Board members were selected due to their level of community influence and their endorsement of mental health care helped to reduce stigma and misconceptions about mental illness in the study site. In a recent trial in Ghana, restraint was not reduced by short-term provision of psychiatric care to people with SMI who were receiving faith healing in prayer camps (Ofori-Atta *et al*., 2018). In our study, the follow-up period was longer, which may have allowed family members and the community to gain confidence in the beneficial effects of treatment and the PRIME intervention incorporated community and system-level interventions as well as task-shared facility-based care.

Alternative approaches to expanding access to care for people with SMI in rural populations in LMICs have been reported. One model is to utilise outreach clinics staffed by mental health specialists who diagnose, prescribe and monitor clinical progress, combined with community-level interventions by non-specialists to promote social inclusion and functional recovery (Srinivasa Murthy *et al*., 2005; Chatterjee *et al*., 2009; Chatterjee *et al*., 2014). Another ‘back-referral’ model is for mental health specialists to provide initial assessment and development of a care plan which is then implemented by non-specialist health workers in the local area combined with task-shared psychosocial interventions (Xiang *et al*., 1994). Augmenting the PRIME model with more intensive and systematic community-based rehabilitation delivered by non-specialists may help to address some of the gaps identified in our study by strengthening engagement with PHC and supporting livelihoods (Asher *et al*., 2016), although the affordability and sustainability of such approaches need evaluation. There are no published reports of these task-sharing service models being successfully, safely and sustainably taken to scale and we have no evidence regarding their relative impact on effective treatment coverage for people with SMI. In the future, randomised controlled trials comparing task-shared models of care for people with SMI are needed to inform policy decisions (Hanlon *et al*., 2016*a*).

### Limitations

PHC workers were informed of the research psychiatric nurse diagnosis and changes in treatment plan. For diagnosis the concordance was high and so this is unlikely to have affected the outcome, but the treatment plan reflected task-shared care with support from a mental health specialist. We only collected data on the positive and not the negative predictive value of the PHC worker diagnosis. Calculating the negative predictive value would be an important focus for future studies. Due to ethical concerns, there was no comparison group of people with SMI receiving ‘usual care’, which would have amounted to no access to evidence-based care for most people. However, outcomes in people with untreated schizophrenia (who formed the majority of our cohort) have been shown to be poor (Ran *et al*., 2001). Given the chronicity and severity of SMI at baseline, spontaneous improvement is unlikely. This is supported by the lack of an association between baseline duration of illness or type of illness onset and outcomes in our study.

## Conclusions

An integrated district level mental health care plan employing a task-sharing approach safely addressed the large treatment gap for people with SMI in a rural, low-income country setting, resulting in improved clinical and social outcomes and reduced human rights abuses. Training and supporting PHC workers to provide mental health care has great potential as a sustainable and feasible approach to the care of people with SMI in resource-poor settings.

## Supplementary material

For supplementary material accompanying this paper visit http://dx.doi.org/10.1017/S2045796019000398. click here to view supplementary material

## Data

The datasets used and/or analysed during the current study are available from the corresponding author on reasonable request. The PRIME datasets will be made publicly available during 2019 (via www.prime.uct.ac.za).

## References

[ref1] American Psychiatric Association (1994) Diagnostic and Statistical Manual of Mental Disorders (4th Edition) (DSM-IV). Washington, DC: APA.

[ref2] Asher L, De Silva M, Hanlon C, Weiss HA, Birhane R, Ejigu DA, Medhin G, Patel V and Fekadu A (2016) Community-based Rehabilitation Intervention for people with Schizophrenia in Ethiopia (RISE): study protocol for a cluster randomised controlled trial. Trials 17, 299. 27342215 10.1186/s13063-016-1427-9 PMC4919867

[ref3] Babor TF, Higgins-Biddle JC, Saunders JB and MG. M (2001) The Alcohol use Disorders Identification Test: Guidelines for use in Primary Care, 2nd Edn. Geneva: World Health Organizarion.

[ref4] Baron EC, Rathod SD, Hanlon C, Prince M, Fedaku A, Kigozi F, Jordans M, Luitel NP, Medhin G, Murhar V, Nakku J, Patel V, Petersen I, Selohilwe O, Shidhaye R, Ssebunnya J, Tomlinson M, Lund C and De Silva M (2018) Impact of district mental health care plans on symptom severity and functioning of patients with priority mental health conditions: the Programme for Improving Mental Health Care (PRIME) cohort protocol. BMC Psychiatry 18, 61. 29510751 10.1186/s12888-018-1642-x PMC5840717

[ref5] Borenstein M, Hedges LV, Higgins JPT and Rothstein HR (2009). Chapter 4: Effect sizes based on means. In Introduction to Meta-Analysis. Chichester, UK: John Wiley & Sons, Ltd, pp. 21–32.

[ref6] Brohan E, Clement S, Rose D, Sartorius N and Thornicroft G (2013) Development and psychometric evaluation of the Discrimination and Stigma Scale (DISC). Psychiatry Research 208, 33–40. 23582210 10.1016/j.psychres.2013.03.007

[ref7] Burlingame GM, Seaman S, Johnson JE, Whipple J, Richardson E, Rees F, Earnshaw D, Spencer R, Payne M and O'Neil B (2006) Sensitivity to change of the Brief Psychiatric Rating Scale – Extended (BPRS-E): an item and subscale analysis. Psychological Services 3, 77–87.

[ref8] Chatterjee S, Pillai A, Jain S, Cohen A and Patel V (2009) Outcomes of people with psychotic disorders in a community-based rehabilitation programme in rural India. British Journal of Psychiatry 195, 433–439. 10.1192/bjp.bp.108.057596 PMC2806571 19880934

[ref9] Chatterjee S, Naik S, John S, Dabholkar H, Balaji M, Koschorke M, Varghese M, Thara R, Weiss HA, Williams P, McCrone P, Patel V and Thornicroft G (2014). Effectiveness of a community-based intervention for people with schizophrenia and their caregivers in India (COPSI): a randomised controlled trial. [Erratum appears in Lancet. 2014 Jun 14;383(9934):2046]. Lancet 383, 1385–1394. 24612754 10.1016/S0140-6736(13)62629-X PMC4255067

[ref10] Dalgard OS, Dowrick C, Lehtinen V, Vazquez-Barquero JL, Casey P, Wilkinson G, Ayuso-Mateos JL, Page H and Dunn G (2006) Negative life events, social support and gender difference in depression: a multinational community survey with data from the ODIN study. Social Psychiatry & Psychiatric Epidemiology 41, 444–451. 16572275 10.1007/s00127-006-0051-5

[ref11] Fekadu A, Hanlon C, Thornicroft G, Lund C, Kaaya S, Alem A, Gureje O, Cleare AJ, Patel V and Prince MJ (2015) Care for bipolar disorder in LMICs needs evidence from local settings. The Lancet Psychiatry 2, 772–773. 26360884 10.1016/S2215-0366(15)00222-9

[ref12] Fekadu A, Hanlon C, Medhin G, Alem A, Selamu M, Giorgis T, Shibre T, Teferra S, Tegegn T, Breuer E, Patel V, Tomlinson M, Thornicroft G, Prince M and Lund C (2016) Development of a scalable mental healthcare plan for a rural district in Ethiopia. British Journal of Psychiatry Supplement 208, s4–s12. 10.1192/bjp.bp.114.153676 PMC4698551 26447174

[ref13] Gureje O, Abdulmalik J, Kola L, Musa E, Yasamy MT and Adebayo K (2015) Integrating mental health into primary care in Nigeria: report of a demonstration project using the mental health gap action programme intervention guide. BMC Health Services Research 15, 242–242. 26094025 10.1186/s12913-015-0911-3 PMC4475323

[ref14] Habtamu K, Alem A, Medhin G, Fekadu A, Dewey M, Prince M and Hanlon C (2017) Validation of the World Health Organization Disability Assessment Schedule in people with severe mental disorders in rural Ethiopia. Health and Quality of Life Outcomes 15, 64. 28381230 10.1186/s12955-017-0647-3 PMC5382515

[ref15] Habtamu K, Alem A, Medhin G, Fekadu A and Hanlon C (2018) Functional impairment among people with severe and enduring mental disorder in rural Ethiopia: a cross-sectional study. Social Psychiatry & Psychiatric Epidemiology 53, 803–814. 10.1007/s00127-00018-01546-00126. 29947862

[ref16] Hailemariam M, Fekadu A, Selamu M, Alem A, Medhin G, Wolde Giorgis T, DeSilva M and Breuer E (2015) Developing a mental health care plan in a low resource setting: the theory of change approach. BMC Health Services Research 15, 429. 26416566 10.1186/s12913-015-1097-4 PMC4587839

[ref18] Hailemichael Y, Hailemariam D, Tirfessa K, Docrat S, Alem A, Medhin G, Lund C, Chisholm D, Fekadu A and Hanlon C (2019) Catastrophic out-of-pocket payments for households of people with severe mental disorder: a comparative study in rural Ethiopia. International Journal of Mental Health Systems 13, 39. 10.1186/s13033-13019-10294-13037. 31164919 PMC6544918

[ref19] Hanlon C, Luitel NP, Kathree T, Murhar V, Shrivasta S, Medhin G, Ssebunnya J, Fekadu A, Shidhaye R, Petersen I, Jordans M, Kigozi F, Thornicroft G, Patel V, Tomlinson M, Lund C, Breuer E, De Silva M and Prince M (2014) Challenges and opportunities for implementing integrated mental health care: a district level situation analysis from five low- and middle-income countries. PLoS One 9, e88437. 24558389 10.1371/journal.pone.0088437 PMC3928234

[ref20] Hanlon C, Medhin G, Selamu M, Breuer E, Worku B, Hailemariam M, Lund C, Prince M and Fekadu A (2015) Validity of brief screening questionnaires to detect depression in primary care in Ethiopia. Journal of Affective Disorders 186, 32–39. 26226431 10.1016/j.jad.2015.07.015

[ref21] Hanlon C, Alem A, Medhin G, Shibre T, Ejigu DA, Negussie H, Dewey M, Wissow L, Prince M, Susser E, Lund C and Fekadu A (2016 *a*) Task sharing for the care of severe mental disorders in a low-income country (TaSCS): study protocol for a randomised, controlled, non-inferiority trial. Trials 17, 76. 26865254 10.1186/s13063-016-1191-x PMC4750210

[ref22] Hanlon C, Fekadu A, Jordans M, Kigozi F, Peterson I, Shidhaye R, Honikman S, Lund C, Prince M, Raja S, Thornicroft G, Tomlinson M and Patel V (2016 *b*) District Mental Health Care Plans for five low- and middle-income countries: commonalities, variations and evidence gaps. British Journal of Psychiatry Supplement 208, s47–s54. 10.1192/bjp.bp.114.153767 PMC4698556 26447169

[ref23] Hanlon C, Alem A, Lund C, Hailemariam D, Assefa E, Giorgis TW and Chisholm D (2019) Moving towards universal health coverage for mental disorders in Ethiopia. International Journal of Mental Health Systems 13, 11. 10.1186/s13033-13019-10268-13039. 30891082 PMC6388484

[ref24] Jordans MJD, Aldridge L, Luitel NP, Baingana F and Kohrt BA (2017) Evaluation of outcomes for psychosis and epilepsy treatment delivered by primary health care workers in Nepal: a cohort study. International Journal of Mental Health Systems 11, 70. 29204183 10.1186/s13033-017-0177-8 PMC5702128

[ref25] Jordans MJD, Luitel NP, Kohrt BA, Rathod SD, Garman EC, De Silva M, Komproe IH, Patel V and Lund C (2019) Community-, facility-, and individual-level outcomes of a district mental healthcare plan in a low-resource setting in Nepal: a population-based evaluation. PLOS Medicine 16, e1002748. 30763321 10.1371/journal.pmed.1002748 PMC6375569

[ref26] Kroenke K and Spitzer RL (2002) The PHQ-9: a new depression diagnostic and severity measure. Psychiatric Annals 32, 509–515.

[ref27] Lauritsen JM and Bruus M (2003). Epidata (Version 3). A comprehensive tool for validated entry and documentation of data. The Epidata Association: Odense, Denmark.

[ref28] McGuffin P, Farmer AE and Harvey I (1991) A polydiagnostic application of operational criteria in studies of psychotic illness: development and reliability of the OPCRIT system. Archives of General Psychiatry 48, 764–770. 1883262 10.1001/archpsyc.1991.01810320088015

[ref29] Ofori-Atta A, Attafuah J, Jack H, Baning F and Rosenheck R (2018) Joining psychiatric care and faith healing in a prayer camp in Ghana: randomised trial. The British Journal of Psychiatry 212, 34–41. 29433613 10.1192/bjp.2017.12

[ref30] Ran MS, Xiang MZ, Huang MS and Shan YH (2001) Natural course of schizophrenia: 2-year follow-up study in a rural Chinese community. British Journal of Psychiatry 178, 154–158. 10.1192/bjp.178.2.154 11157428

[ref31] Sheehan DV, Lecrubier Y, Harnett-Sheehan K, Janavs J, Weiller E, Bonara LI, Keskiner A, Schinka J, Knapp E, Sheehan MF and Dunbar GC (1997) Reliability and validity of the MINI International Neuropsychiatric Interview (M.I.N.I.): according to the SCID-P. European Psychiatry 12, 232–241.

[ref32] Shibre T, Kebede D, Alem A, Negash A, Kibreab S, Fekadu A, Fekadu D, Jacobsson L and Kullgren G (2002) An evaluation of two screening methods to identify cases with schizophrenia and affective disorders in a community survey in rural Ethiopia. International Journal of Social Psychiatry 48, 200–208. 10.1177/002076402128783244 12413248

[ref33] Srinivasa Murthy R, Kishore Kumar KV, Chisholm D, Thomas T, Sekar K and Chandrashekar CR (2005) Community outreach for untreated schizophrenia in rural India: a follow-up study of symptoms, disability, family burden and costs. Psychological Medicine 35, 341–351. 15841870 10.1017/s0033291704003551

[ref34] StataCorp LP (2016) Stata version 13.1. Texas, USA.

[ref35] Üstün TB, Chatterji S, Kostanjsek N, Rehm J, Kennedy C, Epping-Jordan J, Saxena S, von Korffe M, Pullf C and in collaboration with WHO/NIH Joint Project (2010) Developing the world health organization disability assessment schedule 2.0. Bulletin of the World Health Organization 88, 815–823. 21076562 10.2471/BLT.09.067231 PMC2971503

[ref36] Ventevogel P, van de Put W, Faiz H, van Mierlo B, Siddiqi M and Komproe IH (2012) Improving access to mental health care and psychosocial support within a Fragile context: a case study from Afghanistan. PLOS Medicine 9, e1001225. 22666183 10.1371/journal.pmed.1001225 PMC3362640

[ref37] Wang PS, Demler O and Kessler RC (2002) Adequacy of treatment for serious mental illness in the United States. American Journal of Public Health 92, 92–98. 11772769 10.2105/ajph.92.1.92 PMC1447396

[ref38] Wang PS, Angermeyer M, Borges G, Bruffaerts R, Chiu WT, de Girolamo G, Fayyad J, Gureje O, Haro JM, Huang Y, Kessler RC, Kovess V, Levinson D, Nakane Y, Brown MAO, Ormel JH, Posada-Villa J, Aguilar-Gaxiola S, Alonso J, Lee S, Heering S, Pennell B-E, Chatterji S, Ustun TB and for the WHO World Mental Health Survey Consortium (2007) Delay and failure in treatment seeking after first onset of mental health disorders in the world health organization's world mental health survey initiative. World Psychiatry 6, 177–185. 18188443 PMC2174579

[ref39] World Health Organization (2008) Mental Health Gap Action Programme (mhGAP): Scaling up Care for Mental, Neurological, and Substance use Disorders. Geneva: WHO. 26290926

[ref40] World Health Organization (2016) Mental Health Gap Action Programme Intervention Guide (mhGAP-IG) for Mental, Neurological and Substance use Disorders in non-Specialized Health Settings, Version 2.0. Geneva: WHO. 27786430

[ref41] Xiang M, Ran M and Li S (1994) A controlled evaluation of psychoeducational family intervention in a rural Chinese community. British Journal of Psychiatry 154, 544–548. 10.1192/bjp.165.4.544 7804673

