# Supplementary material for: Impact of integrated district level mental health care on clinical and social outcomes of people with severe mental illness in rural Ethiopia: an intervention cohort study
Source: Epidemiol Psychiatr Sci. 2019 Aug 13;29:e45. doi: 10.1017/S2045796019000398 (PMC8061260; doi:10.1017/S2045796019000398)
Supplement: Supplementary file 1 [file S2045796019000398sup001.docx]

**Supplementary 1**

**Characteristics of participants assessed at 12 months compared to those lost to follow-up**

| **Characteristics** | | **Loss to follow-up at 12 months (n=55)**  **N (%)** | **χ^2^ (df) p-value for comparison with people remaining in study (n=245)** |
| --- | --- | --- | --- |
| Sex | Male  Female | 37 (21.5)  18 (14.1) | 2.7198 (1)  P = 0.099 |
| Age (years) | < 20  20 to 29  30 to 39  40 to 49  50 to 59  60 and above | 1 (4.2)  18 (20.2)  17 (21.5)  9 (15.8)  5 (16.7)  5 (23.8) | 4.6878 (5)  P = 0.455 |
| Education | Formal education  No formal education | 34 (18.7)  21 (17.8) | 0.037 (1)  P = 0.847 |
| Marital status | Married  Single, divorced or widowed | 18 (16.2)  37 (19.6) | 0.5275 (1)  P = 0.462 |
| Social support | Intermediate/strong  Poor | 38 (18.4)  17 (18.7) | 0.0044 (1)  P = 0.947 |
| Residence | Urban  Rural | 16 (26.7)  39 (16.3) | 1.0059 (1)  P = 0.316 |
| Socio-economic status | Higher (poverty index ≤ 3)  Lower (poverty index >3) | 35 (19.8)  19 (15.8) | 0.7465 (1)  P = 0.388 |
| Diagnosis | Affective psychosis or bipolar  Non-affective psychosis | 9 (20.5)  46 (18.0) | 0.1550 (1)  P = 0.694 |
| Alcohol use disorder | <8 on AUDIT  ≥8 on AUDIT | 36 (16.9)  19 (21.8) | 1.0059 (1)  P = 0.316 |
| Suicide attempt | No attempt  Attempt in past 3 months | 50 (19.4)  5 (11.9) | 1.3480 (1)  P = 0.289 |
| Restraint | Not restrained  Restrained at baseline | 46 (20.5)  9 (11.8) | 2.8645 (1)  0 = 0.091 |
| Travel time to nearest health facility | ≤ 60 minutes  61 to 120 minutes  ≥121 minutes | 33 (17.2)  13 (21.7)  9 (19.2) | 0.6321 (2)  P = 0.729 |
|  |  | **Mean (SD)** | **t score**  **P-value** |
| Symptom severity | BPRS-E total score | Missing 51.9 (15.5)  Non-missing 47.8 (15.6) | 1.75  P = 0.0812 |
| Disability severity | WHODAS total score | Missing 45.4 (19.0)  Non-missing 42.7 (17.9) | 2.68  P = 0.3291 |
| Depression symptoms | PHQ-9 total score | Missing 14.1 (6.0)  Non-missing 12.6 (5.3) | 1.89  P =0.059 |
|  |  | **Median (IQR)** |  |
| Discrimination | DISC-12 total score | Missing 1 (0, 5)  Non-missing 2 (0, 6) | 1.075 (1)  P = 0.2997 |

**Supplementary file 2:** **Pattern of engagement with primary care mental health care**

**

**

**Supplementary file 3:** **Psychotropic medication prescribed to cohort participants (n=299)**

|  | **Minimum therapeutic dose*** | **Prescribed during follow-up**  **N (%)** | **Median dose (minimum, maximum)**  **in mg** |
| --- | --- | --- | --- |
| Chlorpromazine (oral) | 75mg | 173 (61.9) | 100 (25, 300) |
| Haloperidol (oral) | 1.5mg | 164 (54.8) | 1.5 (1.5, 5) |
| Risperidone (oral) | 2mg | 1 (0.3) | 2 (2, 2) |
| Fluphenazine (depot) | 12.5mg every 4 weeks | 39 (13.0) | 12.5 (12.5, 25) |
| Fluoxetine | 20mg | 15 (5.0) | 20 (20, 20) |
| Amitriptyline | 75mg | 36 (12.0) | 25 (25, 100) |
| Diazepam | - | 12 (4.0) | 5 (5, 10) |

**Supplementary file 4**

**Receipt of community and facility level care over the follow-up period**

| **Over the previous 6 months** | **Time-point T1**  **n (%)** | **Time-point T2**  **n (%)** |
| --- | --- | --- |
| In-patient psychiatric care |  |  |
| Admission to government hospital | 4 (1.6) | 5 (2.0) |
| Out-patient psychiatric care |  |  |
| Consultation with psychiatric nurse or psychiatrist | 23 (9.4) | 18 (7.5) |
| Traditional or religious healing |  |  |
| One or more contact | 32 (13.0) | 28 (11.6) |
| Out-patient care from primary health care workers | 97 (39.6) | 144 (59.8) |
| Main features of out-patient primary health care | (n=97) | (n=144) |
| Assessment | 22 (22.7) | 31 (21.5) |
| Non-specific psychosocial support | 74 (76.3) | 113 (78.5) |
| Prescription of medication | 88 (90.7) | 113 (78.5) |
| Advice regarding medication | 71 (92.2) | 126 (99.2) |
| Explained medication side effects | 69 (89.6) | 123 (98.4) |
| Referral | 0 (0) | 1 (0.7) |
| Community-based support in past 6 months |  |  |
| Received support returning to work | 119 (48.2) | 155 (63.5) |
|  | (n=119) | (n=155) |
| From family | 108 (90.8) | 147 (94.9) |
| From health centre staff | 7 (5.9) | 3 (1.9) |
| From community members/workers | 4 (3.4) | 4 (2.6) |
| Received support with remembering to take medication | 200 (81.0) | 215 (88.1) |
|  | (n=200) | (n=215) |
| From family | 179 (89.5) | 201 (93.9) |
| From health centre staff | 15 (7.5) | 9 (4.2) |
| From community members/workers | 6 (3.0) | 4 (1.9) |
| Received support with improving self-care | 170 (68.8) | 189 (77.5) |
|  | (n=170) | (n=189) |
| From family | 155 (91.2) | 183 (96.8) |
| From health centre staff | 10 (5.9) | 2 (1.1) |
| From community members/workers | 5 (2.9) | 4 (2.1) |
| Received support with meeting people | 17 (6.9) | 11 (4.5) |
| Received support with social engagement | 61 (24.7) | 44 (18.1) |
| Received home visit from health extension worker | 9 (4.4) | 14 (5.8) |

**Supplementary File 5**

**Tests for effect modification of change in primary outcome variables between baseline (T0) and 12 months (T1)**

| **Outcome variables** | **Variables tested for effect modification of change in outcome variable**  **between T0 and T1** | | | | | |
| --- | --- | --- | --- | --- | --- | --- |
|  | **Sex** | **Rural vs. urban residence** | **Distance from health centre > 60 minutes** | **Higher vs. lower Poverty index score** | **Diagnosis: non-affective psychosis vs. affective disorder** | **Higher vs. lower community support at T1** |
| Severe mental disorder symptom severity (BPRS total score) | z = -0.39  p = 0.70 | z = 0.58  p = 0.56 | z = -0.77  p = 0.44 | z = 0.97  p = 0.33 | z = 0.16  p = 0.87 | z = -1.30  p = 0.19 |
| Disability  (WHODAS total score) | z = 0.81  p = 0.42 | z = 0.56  p = 0.58 | z = -1.12  p = 0.26 | z = 0.48  p = 0.63 | z = 1.23  p = 0.22 | z = -1.69  p = 0.09 |

**Supplementary File 6**

**Tests for effect modification of change in primary outcome variables between baseline (T0) and 12 months (T2)**

| **Outcome variables** | **Variables tested for effect modification of change in outcome variable**  **between T0 and T2** | | | | | | |
| --- | --- | --- | --- | --- | --- | --- | --- |
|  | **Sex** | **Rural vs. urban residence** | **Distance from health centre > 60 mins** | **Higher vs. lower Poverty index score** | **Diagnosis: non-affective psychosis vs. affective disorder** | **Minimally adequate treatment from T0 to T2** | **Higher vs. lower community support at from T0 to T2** |
| Severe mental disorder symptom severity (BPRS total score) | z = -0.79  p = 0.43 | z = 1.56  p = 0.12 | z = 0.86  p = 0.39 | z = 0.87  p = 0.38 | z = -0.19  p = 0.85 | z = 0.09  p = 0.93 | z = 0.76  p = 0.45 |
| Disability  (WHODAS total score) | z = -1.07  p = 0.29 | z = 0.46  p = 0.65 | z = -0.72  p = 0.47 | z = -0.34  p = 0.73 | z = -0.11  p = 0.91 | z = -1.20  p = 0.23 | z = -0.91  p = 0.36 |
